# Trends, outcomes and patient- and hospital-level factors associated with sepsis following cesarean delivery: a propensity-matched nationwide inpatient analysis (2016–2022)

**DOI:** 10.3389/fsurg.2026.1871936

**Published:** 2026-10-21

**Authors:** Lanhui Lan, Xueliang Liu, Xianghua Cao, Lili Zheng, Fengshan Gong, Jian Wang, Qinfeng Yang, Xueping Li, Haoran Yan

**Affiliations:** 1 Department of Anesthesiology, Dongguan Tungwah Hospital, Dongguan, China; 2 Dongguan Key Laboratory of Anesthesia and Enhanced Recovery After Surgery, Dongguan Tungwah Hospital, Dongguan, Guangdong, China; 3 Department of Anesthesiology, Dongguan SongShan Lake Tungwah Hospital, Dongguan, China; 4 Division of Orthopaedic Surgery, Department of Orthopaedics, Nanfang Hospital, Southern Medical University, Guangzhou, Guangdong, China

**Keywords:** cesarean section, factors associated with sepsis, hospitalization costs, length of stay, mortality, nationwide inpatient sample, propensity score matching, sepsis

## Abstract

**Background:**

Sepsis is a major global health concern and a serious complication of cesarean delivery. However, contemporary nationwide data describing both the temporal trend in post-cesarean sepsis and its downstream clinical and economic burden—particularly in propensity-matched comparisons—remain limited.

**Methods:**

We conducted a retrospective, propensity-matched analysis of all adult inpatients undergoing cesarean delivery recorded in the U.S. Nationwide Inpatient Sample (NIS, 2016–2022). Sepsis and cesarean delivery were defined using International Classification of Diseases, Tenth Revision, Clinical Modification (ICD-10-CM) codes. After 1:5 propensity-score matching on demographics, comorbidity burden and hospital characteristics, we compared in-hospital mortality, length of stay (LOS), total hospitalization costs, and the incidence of perioperative complications between septic and non-septic patients. Multivariable logistic regression was used to identify patient- and hospital-level factors independently associated with sepsis.

**Results:**

Of 1,619,187 hospitalizations for cesarean delivery, 1,589,186 adults met the inclusion criteria. Annual sepsis prevalence rose from 0.058% in 2016 to 0.136% in 2022. In the 1:5 propensity-matched cohort (*n* = 10,050), septic patients had markedly higher in-hospital mortality (2.4% vs. 0.0%, *P* < 0.001), longer median LOS (6 vs. 3 days, *P* < 0.001), and approximately 2.6-fold higher median total hospitalization costs (USD 71,466 vs. USD 27,063, *P* < 0.001). Non-elective admission, treatment in teaching hospitals, a higher comorbidity burden, Asian/Pacific Islander race, and hospital region were independently associated with sepsis. After propensity matching, septic patients also showed substantially elevated adjusted odds of pulmonary embolism (OR 61.7), postoperative shock (OR 28.7), deep-vein thrombosis (OR 24.6), urinary tract infection (OR 25.5), and chronic respiratory diseases (OR 13.2).

**Conclusions:**

In this large U.S. study, sepsis after cesarean section was linked to much higher death rates, twice the hospital stay, about 2.6 times higher costs, and more severe problems like blood clots, shock, infections, and heavy bleeding. These results show that post-cesarean section sepsis is a growing concern, remind us to spot at-risk mothers early, and offer a benchmark against which prevention and cost-effective care can be evaluated.

## Introduction

Over the last two decades, global cesarean section (CS) rates have risen steadily, driven by evolving fertility attitudes, advances in fetal monitoring, and a widening range of obstetric indications (1). In the United States, nationally representative data show CS rates well above the 10%–15% range considered medically optimal, and recent surveys report rates of approximately one-third of all deliveries (2). Globally, CS remains the most commonly performed inpatient major surgical procedure and is associated with disproportionately higher rates of postoperative morbidity than vaginal delivery (3). Infectious complications are among the most frequent and the most preventable adverse events following CS. Approximately one in ten women develops a post-CS surgical-site infection (SSI), with rates varying substantially across regions, payer systems and case-mix (4–7). Beyond SSI, postpartum infections include endometritis, urinary tract infection (UTI), wound dehiscence, and septicemia, which collectively now constitute a substantial share of severe maternal morbidity (SMM) and a recognized target for quality improvement (8–11). Prophylactic antibiotic regimens and standardized intra-operative protocols are well established for preventing SSI, but their effects on the downstream cascade of sepsis, organ dysfunction and intensive-care utilization are less well characterized (12, 13).

Infection is one of the leading preventable causes of maternal mortality, with pregnancy-related sepsis accounting for approximately 11% of maternal deaths (14, 15). Estimates of maternal sepsis incidence vary widely—from less than 0.1% in high-income settings to several percent in low- and middle-income countries (LMICs)—reflecting differences in case definitions, surveillance intensity and underlying obstetric risk profile (16, 17). In the United States specifically, the burden of maternal sepsis has been rising, with longitudinal analyses showing persistent increases in postpartum infection rates and in SMM driven by infection-related causes (8, 18). Sepsis following CS is best understood not as a single event but as a clinical syndrome with heterogeneous downstream complications, the most clinically important of which include septic shock, multiple-organ dysfunction, acute kidney injury, coagulopathy and venous thromboembolism (VTE)—particularly pulmonary embolism (PE) and deep-vein thrombosis (DVT)—all exacerbated by the hypercoagulable state of pregnancy and the post-operative immobilization that often accompanies severe sepsis (19–22). Endothelial dysfunction and microcirculatory failure are now considered central drivers of organ injury in sepsis, providing a unifying biological explanation for the cardiovascular, respiratory, renal and hematological complications observed in obstetric patients (23–25), with pulmonary complications, electrolyte and acid-base disturbances and fluid overload as additional common contributors to morbidity in this population (26–29).

Beyond clinical outcomes, sepsis imposes a substantial economic and resource-utilization burden. Patients who develop sepsis after major surgery consistently experience longer intensive-care and total hospital stays and higher total hospitalization costs than matched non-septic controls, with estimates of excess median length of stay (LOS) ranging from 3 to 7 days and excess hospital charges commonly exceeding USD 30,000 per admission (30, 31); in the broader U.S. sepsis population, the attributable in-hospital cost exceeds USD 20 billion annually (32). However, contemporary nationwide estimates specific to post-cesarean sepsis in the United States remain scarce, and few studies have simultaneously quantified temporal trends, propensity-matched clinical outcomes, and patient- and hospital-level determinants within a single cohort. To address this knowledge gap, the present study used the Nationwide Inpatient Sample (NIS)—the largest publicly available all-payer inpatient database in the United States, maintained by the Healthcare Cost and Utilization Project (HCUP) of the Agency for Healthcare Research and Quality (AHRQ).

## Methods

### Data source and setting

For this analysis, we used data from the Nationwide Inpatient Sample (NIS) for the years 2016–2022. The NIS is sponsored by the Agency for Healthcare Research and Quality (AHRQ) through the Healthcare Cost and Utilization Project (HCUP) and contains approximately 7 million unweighted discharges per year from a stratified sample of ∼20% of U.S. community hospitals. Because NIS records represent discharges—not individual patients—each hospitalization is treated as a unique observation (33). The NIS uses a complex two-stage sampling design (first stage: stratified random selection of hospitals; second stage: systematic random selection of discharges) and provides survey discharge weights (DISCWT) that allow generation of national estimates. Beginning with the 2016 redesign, the NIS samples discharges from all participating hospitals (rather than all discharges from a subset of hospitals), preserving national representativeness. All analyses therefore incorporate DISCWT to obtain population-level estimates (34). Because the NIS contains only de-identified data, this study was exempt from institutional review board approval and informed consent. Data analysis was performed under a standard HCUP Data Use Agreement; authors had no direct patient contact.

### Study population, inclusion and exclusion criteria

This study utilized data from the Nationwide Inpatient Sample (NIS) database spanning January 1, 2016, through December 31, 2022. The initial cohort comprised 1,619,187 hospitalizations for cesarean delivery, identified by the presence of any of the following ICD-10-CM procedure codes in any procedure field: 10D00Z0 (extraction of products of conception, high, open), 10D00Z1 (low, open), or 10D00Z2 (extraperitoneal, open). To optimize diagnostic specificity for maternal sepsis following cesarean section, sepsis cases were restricted to hospitalizations bearing both a cesarean procedure code and at least one sepsis diagnosis code (8, 18); cesarean hospitalizations without a sepsis code formed the comparison group. Exclusion criteria were applied sequentially as follows: (1) admission age < 18 years; (2) missing data for DISCWT or other essential survey-design variables; (3) discharge disposition incompatible with an uncomplicated delivery hospitalization (e.g., transfer to another acute-care facility); and (4) records with biologically implausible age–sex combinations. Following these exclusions—which removed 10,474 patients younger than 18 years, 15,236 with incomplete records and 4,291 meeting the remaining exclusion criteria (3)-(4)—1,589,186 unweighted hospitalizations remained for analytical consideration (Figure 1). This final pre-match cohort was then stratified into a sepsis group (*n* = 1,675) and a non-sepsis group (*n* = 1,587,511). To mitigate confounding by baseline covariates, we performed propensity score matching at a 1:5 ratio using key demographic and clinical characteristics, yielding a final analytic cohort of 1,675 sepsis cases and 8,375 matched controls (Figure 1).

**Figure 1:**
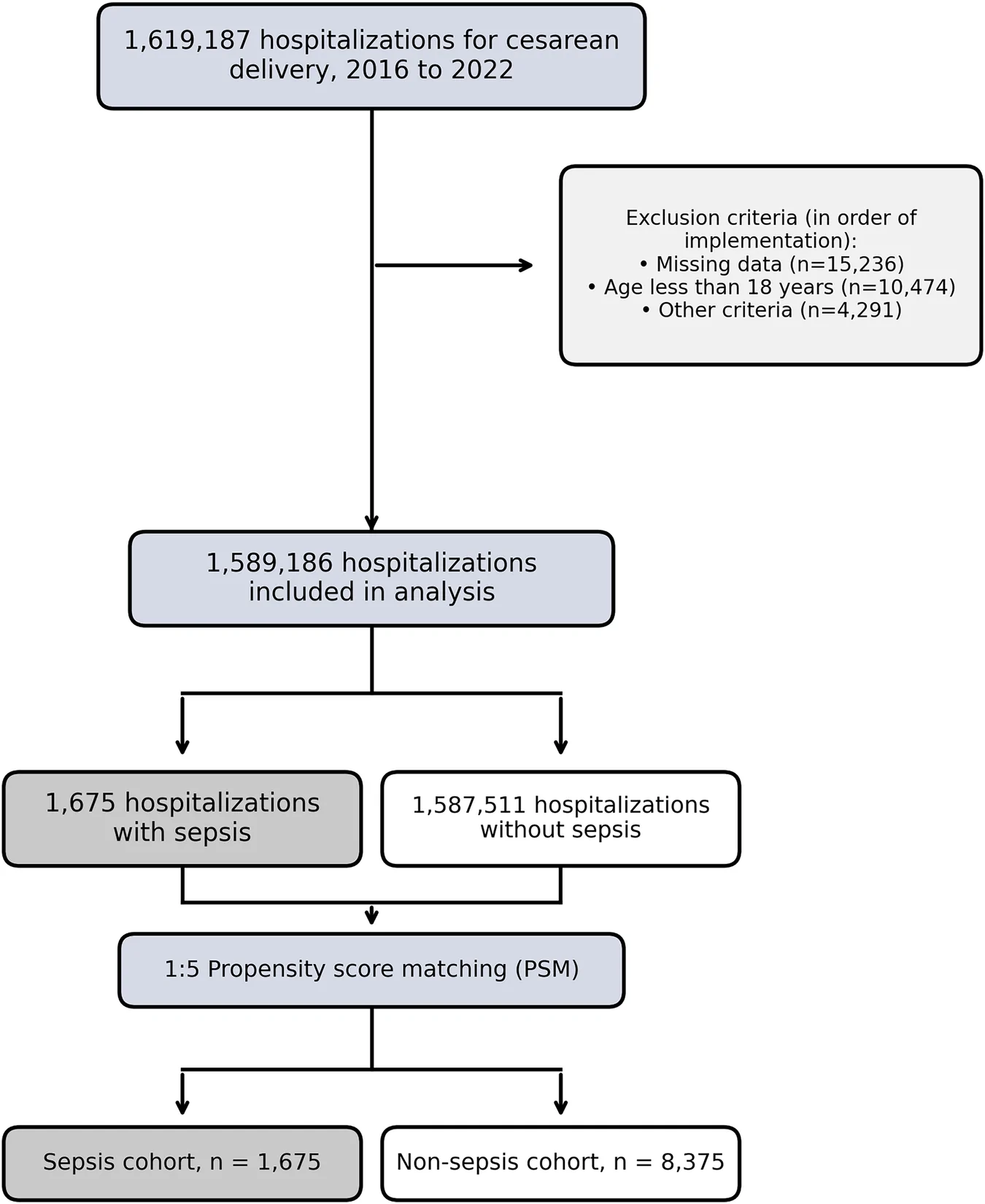
Flow diagram of cohort selection and propensity-score matching for the nationwide inpatient sample (NIS). Of 1,619,187 hospitalizations for cesarean delivery identified in the 2016-2022 U.S. Nationwide Inpatient Sample, 15,236 records were excluded for missing data and 10,474 for patient age below 18 years, and 4,291 for other exclusion criteria, leaving 1,589,186 hospitalizations in the primary analytic cohort. Within this cohort, 1,675 patients had a discharge diagnosis of sepsis and 1,587,511 did not. Following 1:5 propensity-score matching on demographics, comorbidity burden and hospital characteristics, the final analytic sample comprised 1,675 patients with sepsis and 8,375 matched patients without sepsis (total matched *n*=10,050), with between-group standardized mean differences < 0.10 for all matched covariates, indicating adequate balance.

### Exposure, outcomes and covariates

Exposure was defined as sepsis occurring during the cesarean delivery hospitalization, identified by ICD-10-CM codes A40.x (streptococcal sepsis), A41.x (other sepsis), R65.20 (severe sepsis without septic shock), or R65.21 (severe sepsis with septic shock), recorded as any diagnosis field (Supplementary Tables S1, S2). The primary pre-specified outcomes of interest were the temporal trend in sepsis prevalence (2016–2022) and the patient- and hospital-level factors independently associated with sepsis in the multivariable logistic model. The secondary pre-specified outcomes, compared between septic and propensity-matched non-septic patients, were (a) in-hospital mortality, (b) length of stay (LOS, days), (c) total hospitalization cost (TOTCHG, USD), and (d) a pre-specified panel of perioperative complications (Supplementary Table S3).

Covariates were selected *a priori* based on clinical relevance and consistent with established NIS methodological standards (35). Patient-level covariates included age (categorical: 18–24, 25–29, 30–34, ≥35 years), race (White, Black, Hispanic, Asian/Pacific Islander, Native American, Other), primary payer (Medicare, Medicaid, Private, Self-pay, No charge, Other), elective vs. non-elective admission, and the number of chronic comorbidities (categorized 0, 1, 2, ≥3 from a 17-item Elixhauser-style list; Supplementary Table S4 summarizes univariate and multivariable associations for each comorbidity). Hospital-level covariates included bed size (small, medium, large), region (Northeast, Midwest/North Central, South, West), teaching status, and urban location.

Complications (e.g., congestive heart failure, pulmonary embolism, deep-vein thrombosis, postoperative shock, urinary tract infection, postpartum hemorrhage, chronic respiratory disease, blood transfusion, fluid and electrolyte disorders, amniotic fluid embolism) were identified using ICD-10-CM codes in any secondary diagnosis position and are summarized in Supplementary Tables S3, S5.

### Statistical analysis

All analyses were performed using IBM SPSS Statistics v25 and R v4.3.0, accounting for the NIS complex survey design (stratification, clustering and DISCWT) per HCUP recommendations (33, 35). Continuous variables (LOS, TOTCHG) are reported as medians with interquartile ranges (IQR) and compared using Wilcoxon rank-sum tests with the Rao–Scott correction. Categorical variables are reported as counts (weighted %) and compared using Pearson *χ*^2^ tests with the second-order Rao–Scott correction. All statistical tests were two-sided.

To reduce confounding by indication and baseline risk, we performed 1:5 propensity-score matching (PSM) between septic and non-septic patients using nearest-neighbor matching without replacement and a caliper width of 0.2 × SD of the logit of the propensity score (36). The propensity score was estimated from a logistic model that included all pre-specified patient- and hospital-level covariates. Covariate balance before and after matching was assessed using standardized mean differences (SMD), with SMD < 0.10 indicating adequate balance (Table 1 reports the SMDs and *p*-values after matching). After matching, outcomes were compared using paired Wilcoxon signed-rank tests for continuous variables and McNemar's tests for categorical variables. Associations between sepsis and each pre-specified complication were quantified using multivariable logistic regression (adjusting for the same covariates as above); results are reported as odds ratios (OR) with 95% confidence intervals (CI) and visualized in Figure 2 and tabulated in Supplementary Table S5. The pre-specified primary significance threshold was *p* < 0.01 (Bonferroni-corrected for multiple secondary outcomes). All CIs are reported at the 95% level.

**Figure 2:**
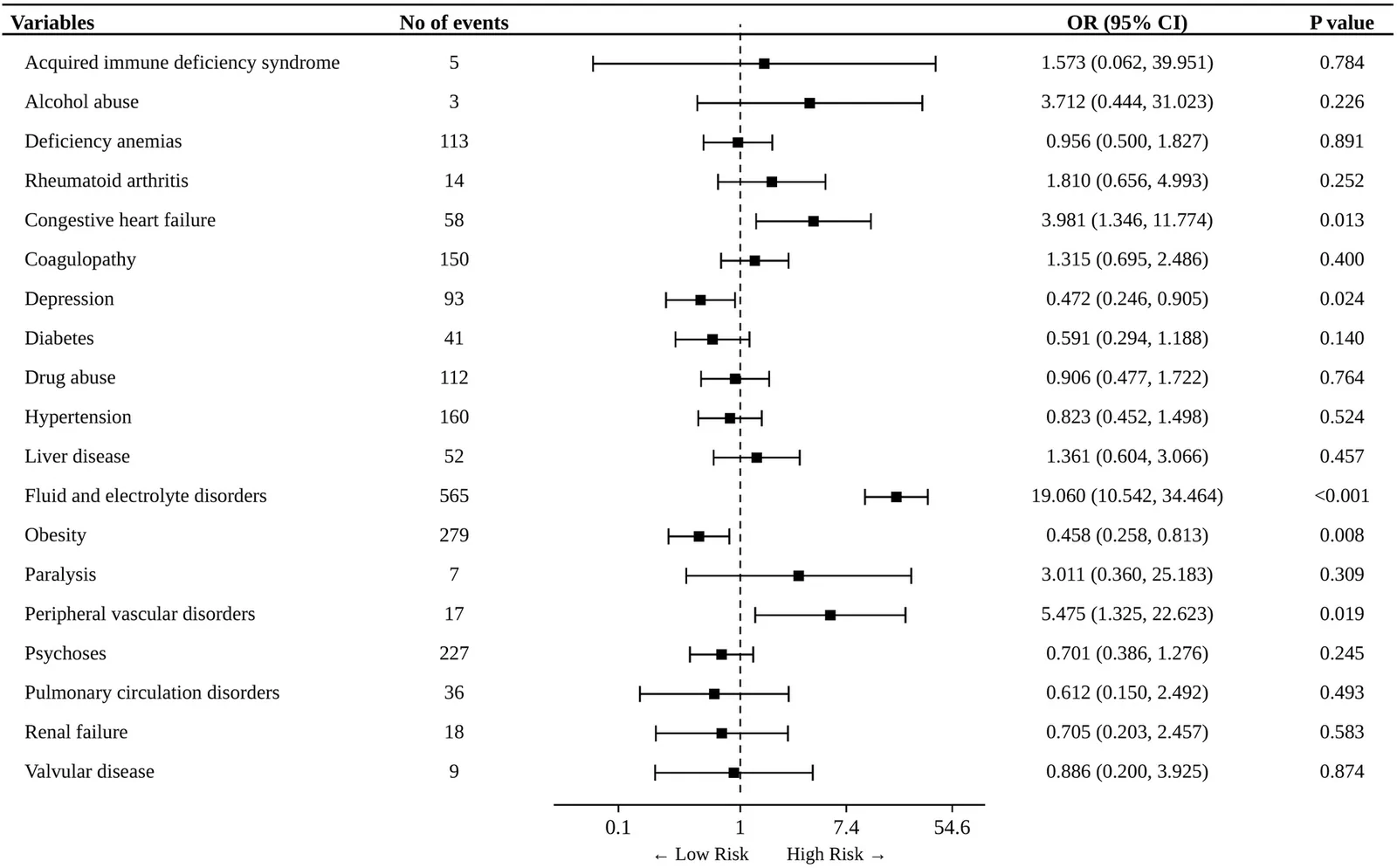
Association between sepsis and preoperative comorbidities after propensity matching. Forest plot of adjusted odds ratios (aOR) with 95% confidence intervals (CI) and *p*-values for preoperative comorbidities in septic vs. matched non-septic patients, derived from multivariable logistic regression adjusting for age, race, primary payer, comorbidity count, elective admission, hospital bed size, region, teaching status and urban location. Squares indicate point estimates and horizontal lines indicate 95% CIs; the dashed vertical line marks OR=1 (log scale). The aORs (95% CI) were 3.98 (1.35-11.8; *p* = 0.013) for congestive heart failure and 19.06 (10.5−34.5; *p* < 0.001) for fluid and electrolyte disorders.

**Table 1:** Baseline characteristics and outcomes of patients with and without sepsis after cesarean section, before and after propensity score matching, 2016–2022.

Characteristics	Sepsis	No Sepsis	P	Matched-No Sepsis	SMD/P
Total (*n* = count)	1,675	1,587,511		8,375	
Total incidence (%)	0.1%				
Age (median, years)	29 (25–34)	30 (26–34)	<0.001	29 (25–33)	<0.001
Age group (%)					
18–24	415 (24.8%)	275,970 (17.4%)	<0.001	1,914 (22.9%)	0.011^S^
25–29	425 (25.4%)	428,181 (27.0%)		2,430 (29.0%)	
30–34	476 (28.4%)	498,354 (31.4%)		2,354 (28.1%)	
≥35	359 (21.4%)	385,006 (24.3%)		1,677 (20.0%)	
Race (%)					
White	564 (33.7%)	785,062 (49.5%)	<0.001	3,311 (39.5%)	0.006^S^
Black	347 (20.7%)	252,463 (15.9%)		1,330 (15.9%)	
Hispanic	438 (26.1%)	320,307 (20.2%)		2,088 (24.9%)	
Asian or Pacific Islander	158 (9.4%)	92,571 (5.8%)		564 (6.7%)	
Native American	15 (0.9%)	10,549 (0.7%)		69 (0.8%)	
Other	153 (9.1%)	126,559 (8.0%)		1,013 (12.1%)	
Number of Comorbidity (%)					
0	597 (35.6%)	1,088,961 (68.6%)	<0.001	5,700 (68.1%)	<0.001
1	543 (32.4%)	344,540 (21.7%)		1,859 (22.2%)	
2	290 (17.3%)	115,550 (7.3%)		614 (7.3%)	
≥3	245 (14.6%)	38,460 (2.4%)		202 (2.4%)	
Type of insurance (%)					
Medicare	30 (1.8%)	13,107 (0.8%)	<0.001	90 (1.1%)	0.002^S^
Medicaid	871 (52.0%)	645,162 (40.6%)		4,190 (50.0%)	
Private insurance	699 (41.7%)	848,396 (53.4%)		3,833 (45.8%)	
Self-pay	36 (2.1%)	37,202 (2.3%)		165 (2.0%)	
No charge	4 (0.2%)	1,036 (0.1%)		6 (0.1%)	
Other	35 (2.1%)	42,608 (2.7%)		91 (1.1%)	
Bed size of hospital (%)					
Small	211 (12.6%)	298,438 (18.8%)	<0.001	1,000 (11.9%)	0.0017^S^
Medium	413 (24.7%)	478,038 (30.1%)		2,274 (27.2%)	
Large	1,051 (62.7%)	811,035 (51.1%)		5,101 (60.9%)	
Region of hospital (%)					
Northeast	221 (13.2%)	260,790 (16.4%)	<0.001	964 (11.5%)	−0.005^S^
Midwest or North Central	314 (18.7%)	310,135 (19.5%)		1,378 (16.5%)	
South	610 (36.4%)	681,603 (42.9%)		3,812 (45.5%)	
West	530 (31.6%)	334,983 (21.1%)		2,221 (26.5%)	
Elective admission (%)	538 (32.1%)	906,106 (57.1%)	<0.001	4,656 (55.6%)	<0.001
Type of hospital (teaching %)	1,402 (83.7%)	1,153,945 (72.7%)	<0.001	5,984 (71.5%)	<0.001
Location of hospital (urban, %)	1,614 (96.4%)	1,452,499 (91.5%)	<0.001	7,637 (91.2%)	<0.001
Died (%)	41 (2.4%)	267 (0.0%)	<0.001	0 (0.0%)	<0.001
LOS (median, d)	6 (4–10)	3 (2–4)	<0.001	3 (2–4)	<0.001
TOTCHG (median, $)	71,466 (43,574–134,893)	25,920 (18,134–37,828)	<0.001	27,063 (18,865–39,041)	<0.001

LOS, length of stay; TOTCHG, total hospital charge; SMD, standardized mean difference. Continuous variables are presented as median (interquartile range) and categorical variables as *n* (%). Patients with sepsis were matched 1:5 to patients without sepsis. *P* values compare the sepsis group with the unmatched and the matched no-sepsis groups, respectively; in the matched comparison, values marked with superscript S are standardized mean differences (SMD).

**Figure 3:**
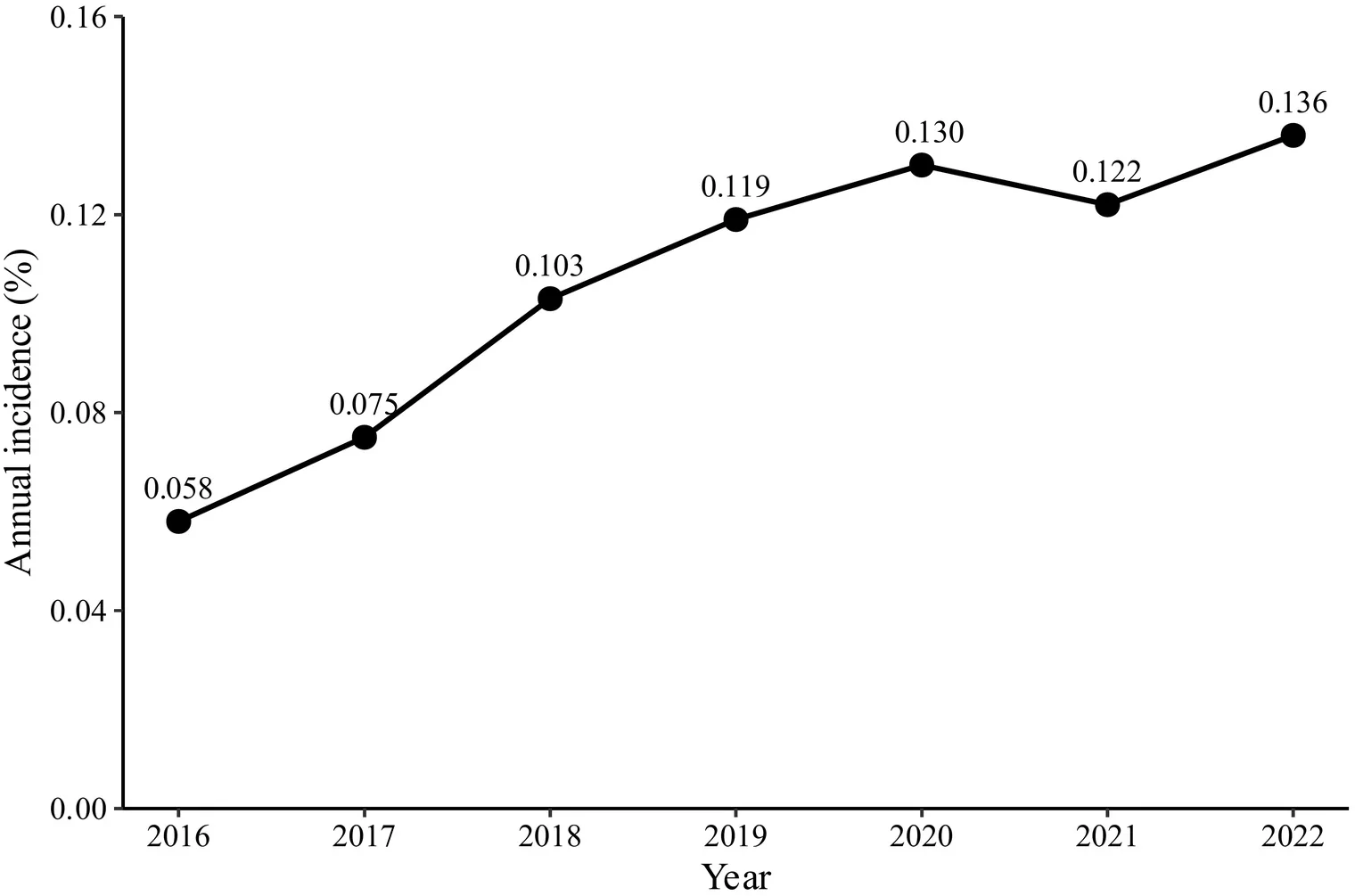
Trends in sepsis prevalence among cesarean delivery inpatients (2016−2022). The line plot shows the weighted annual prevalence of sepsis (%, *y*-axis) among 1,589,186 adult hospitalizations for cesarean delivery recorded in the U.S. Nationwide Inpatient Sample between 2016 and 2022 (*x*-axis). Annual point estimates were 0.058% (2016), 0.075% (2017), 0.103% (2018), 0.119% (2019), 0.130% (2020), 0.122% (2021) and 0.136% (2022), corresponding to an approximately 2.3-fold relative increase over the seven-year window (Cochran-Armitage *p* < 0.001).

## Results

### Sepsis hospitalizations trends in cesarean section patients from 2016 to 2022

From 2016 to 2022, the weighted annual prevalence of sepsis among 1,589,186 adult inpatients hospitalized for cesarean delivery increased from 0.058% in 2016 to 0.136% in 2022—an absolute increase of 0.078 percentage points and a relative increase of approximately 135% over the seven-year study window. Year-on-year point estimates are: 2016, 0.058%; 2017, 0.075%; 2018, 0.103%; 2019, 0.119%; 2020, 0.130%; 2021, 0.122%; 2022, 0.136% (Figure 3; Supplementary Table S6). Over the seven-year observation period, the cumulative sepsis incidence following cesarean delivery was 0.100% (Table 1).

### Patient characteristics before and after propensity-score matching

Before matching, septic patients (*n* = 1,675; weighted frequency) differed substantially from non-septic patients (*n* = 1,587,511) across virtually every baseline characteristic (Table 1; all *p* < 0.001). Compared with non-septic patients, septic patients were younger (median 29 vs. 30 years; 18-24 years: 24.8% vs. 17.4%; ≥35 years: 21.4% vs. 24.3%), more likely to be non-White (particularly Asian/Pacific Islander, 9.4% vs. 5.8%), more likely to be Medicaid-insured (52.0% vs. 40.6%), less likely to be electively admitted (32.1% vs. 57.1%), and more likely to be treated at large (62.7% vs. 51.1%), teaching (83.7% vs. 72.7%) and urban (96.4% vs. 91.5%) hospitals. They also carried a markedly higher comorbidity burden: only 35.6% had zero comorbidities (vs. 68.6% in non-septic patients), 17.3% had two comorbidities, and 14.6% had ≥ 3 (vs. 7.3% and 2.4%, respectively). After 1:5 propensity-score matching, all between-group standardized mean differences were below 0.10 (Table 1), indicating adequate balance on the matched covariates. In the multivariable logistic regression on the full unmatched cohort (Table 2), independent factors associated with sepsis were elective admission (adjusted OR 0.47, 95% CI 0.40-0.54, *p* < 0.001; i.e., non-elective admission was associated with higher odds of sepsis), treatment at a teaching hospital (OR 1.55, 95% CI 1.25–1.92, *p* < 0.001), Asian/Pacific Islander race (OR 1.61, 95% CI 1.22–2.12, *p* = 0.001), higher comorbidity burden (1 comorbidity: OR 2.25, 95% CI 1.28–3.96; 2 comorbidities: OR 3.68, 95% CI 1.22–11.09; ≥3 comorbidities: OR 8.70, 95% CI 1.45–52.12, all *p* < 0.05), hospitalization in the South region (OR 0.65 vs. Northeast, 95% CI 0.51–0.82, *p* < 0.001), and “other” primary payer (OR 3.83, 95% CI 1.50–9.80, *p* = 0.005).

**Table 2:** Multivariable logistic regression analysis of factors associated with sepsis after cesarean section in the full (unmatched) cohort.

Variable	Multivariate Logistic Regression		
	OR	95% CI	P
Age group			
18–24	Ref	——	——
25–29	0.697	0.568–0.855	0.001
30–34	0.781	0.636–0.960	0.019
≥35	0.701	0.558–0.881	0.002
Race			
White	Ref	——	——
Black	1.189	0.954–1.481	0.123
Hispanic	1.200	0.980–1.469	0.078
Asian or Pacific Islander	1.611	1.222–2.124	0.001
Native American	1.377	0.650–2.917	0.404
Other	0.824	0.631–1.077	0.156
Number of Comorbidity			
0	Ref	——	——
1	2.247	1.275–3.963	0.005
2	3.679	1.221–11.086	0.021
≥3	8.700	1.452–52.118	0.018
Type of insurance			
Medicare	Ref	——	——
Medicaid	1.364	0.621–2.996	0.440
Private insurance	1.646	0.749–3.617	0.214
Self-pay	1.927	0.763–4.868	0.165
No charge	4.359	0.703–27.007	0.114
Other	3.828	1.495–9.802	0.005
Bed size of hospital			
Small	Ref	——	——
Medium	0.843	0.657–1.082	0.180
Large	0.893	0.714–1.116	0.318
Region of hospital			
Northeast	Ref	——	——
Midwest or North Central	0.983	0.752–1.284	0.899
South	0.648	0.510–0.822	<0.001
West	1.113	0.874–1.416	0.385
Elective admission	0.468	0.402–0.544	<0.001
Type of hospital (teaching)	1.549	1.251–1.917	<0.001
Location of hospital (urban)	1.247	0.842–1.846	0.271

OR, odds ratio; CI, confidence interval; Ref, reference. Results are presented as odds ratios (OR) with 95% confidence intervals (CI). Ref indicates the reference category.

### Postoperative outcomes in the propensity-matched cohort

After 1:5 propensity-score matching (matched *n* = 10,050: 1,675 septic and 8,375 non-septic) (Figure 1), all three pre-specified secondary outcomes differed significantly between groups (Table 1). Median LOS was 6 days (IQR 4–10) in septic patients vs. 3 days (IQR 2–4) in matched non-septic patients (paired Wilcoxon signed-rank *p* < 0.001), corresponding to an absolute increase of 3 days or a doubling of the typical post-CS hospitalization. Median total hospitalization costs were USD 71,466 (IQR 43,574–134,893) in septic patients vs. USD 27,063 (IQR 18,865–39,041) in non-septic patients (paired Wilcoxon *p* < 0.001), representing approximately 2.6-fold higher median costs—an absolute excess of USD 44,403 per admission. In-hospital mortality was 2.4% (*n* = 41) among septic patients vs. 0.0% in the matched non-septic comparator (McNemar *p* < 0.001)(Table 1). All three comparisons remained highly significant after Bonferroni correction for multiple comparisons (threshold *p* < 0.01).

### Sepsis-associated perioperative complications

In multivariable logistic regression adjusted for the same covariates as above, septic patients had markedly higher adjusted odds of multiple severe perioperative complications (Figures 2, 4; Supplementary Tables S4, S5). The largest effect sizes were observed for pulmonary embolism (adjusted OR 61.7, 95% CI 6.7–566.6, *p* < 0.001), postoperative shock (OR 28.7, 95% CI 6.3–131.3, *p* < 0.001), deep-vein thrombosis (OR 24.6, 95% CI 4.7–129.7, *p* < 0.001), urinary tract infection (OR 25.5, 95% CI 12.7–51.5, *p* < 0.001) and fluid and electrolyte disorders (OR 19.1, 95% CI 10.5-34.5, *p* < 0.001). Smaller but still significant associations were observed for chronic respiratory diseases (OR 13.2, 95% CI 2.9–59.2, *p* = 0.001), blood transfusion (OR 3.85, 95% CI 2.90–5.11, *p* < 0.001), postpartum hemorrhage (OR 2.36, 95% CI 1.83–3.04, *p* < 0.001) and congestive heart failure (OR 3.98, 95% CI 1.35–11.8, *p* = 0.013) (Supplementary Tables S4, S5).

**Figure 4:**
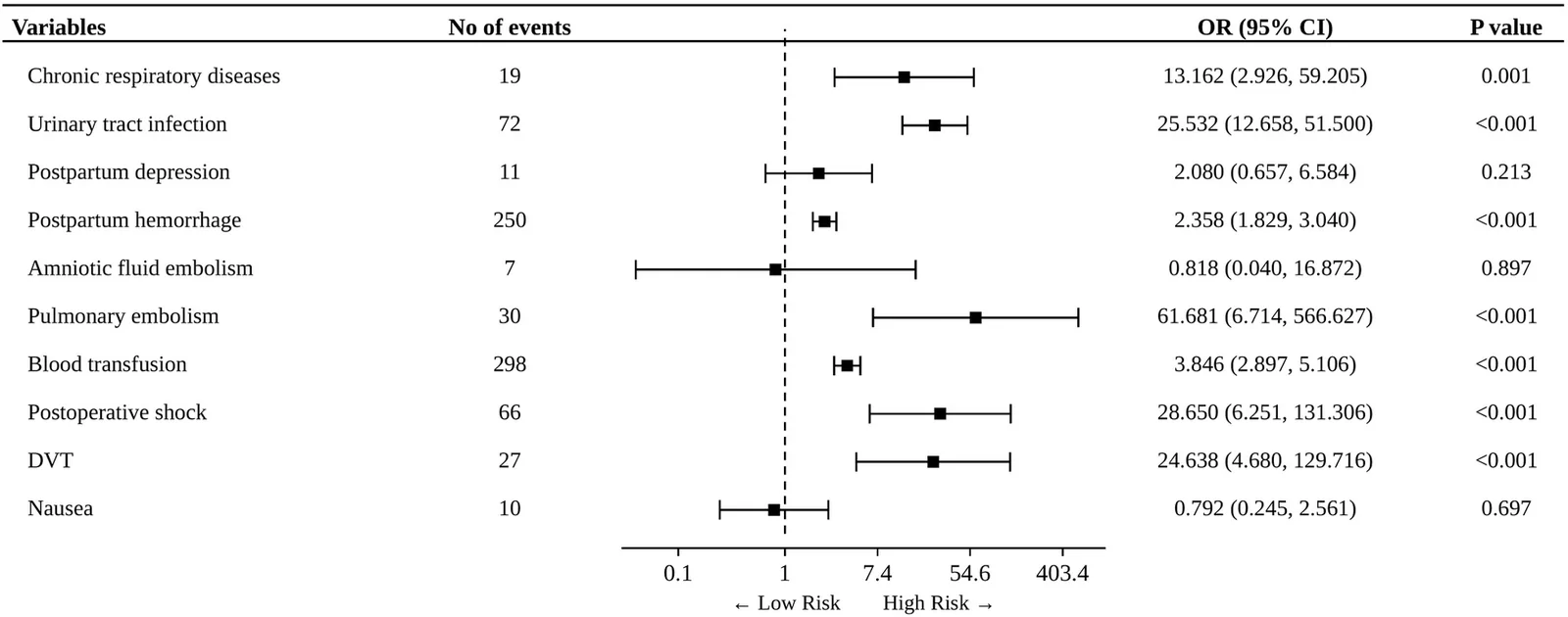
Increased risks of key postoperative complications after propensity matching. Forest plot of adjusted odds ratios (aOR) with 95% confidence intervals (CI) and *p*-values for postoperative complications in septic vs. matched non-septic patients, derived from multivariable logistic regression adjusting for age, race, primary payer, comorbidity count, elective admission, hospital bed size, region, teaching status and urban location. Squares indicate point estimates and horizontal lines indicate 95% CIs; the dashed vertical line marks OR=1 (log scale). The aORs (95% CI) were: chronic respiratory diseases 13.16 (2.93−59.2), urinary tract infection 25.53 (12.7−51.5), postpartum hemorrhage 2.36 (1.83−3.04), pulmonary embolism 61.68 (6.71−566.6), blood transfusion 3.85 (2.90−5.11), postoperative shock 28.65 (6.25−131.3) and deep-vein thrombosis (DVT) 24.64 (4.68−129.7). All *p* < 0.001 except chronic respiratory diseases (*p* = 0.001).

## Discussion

### Principal findings

In this propensity-matched, contemporary nationwide US analysis of 1,589,186 hospitalizations for cesarean delivery (2016–2022), we found that sepsis—although uncommon at the individual-patient level (weighted prevalence 0.058%–0.136%)—was independently associated with substantially worse clinical and economic outcomes. After 1:5 propensity-score matching, septic patients experienced: in-hospital mortality that was higher than in non-septic patients (2.4% vs. 0.0%); twice the median hospital length of stay (6 vs. 3 days); approximately 2.6-fold higher median total hospitalization costs (USD 71,466 vs. USD 27,063); and were markedly associated with several life-threatening complications, with the largest effect sizes for pulmonary embolism, postoperative shock, deep-vein thrombosis, urinary tract infection and fluid/electrolyte disorders (Figures 2 and 4).

### Incidence and mortality: benchmarking against high-income countries

The 2016–2022 weighted prevalence of post-cesarean sepsis in our cohort (0.058%–0.136%, i.e., 5.8–13.6 per 10,000 cesarean hospitalizations) is higher than recent all-delivery estimates from comparable settings: 3.7 per 10,000 deliveries in 2015 in an earlier analysis of the same database (NIS 2002–2015, sepsis defined as infection with end-organ dysfunction) (37); 3.8 per 10,000 deliveries (0.038%) within 42 days of delivery discharge in the U.S. National Readmissions Database (2013–2016) (38); and 4.7 per 10,000 maternities for severe maternal sepsis in the UK national obstetric surveillance system (2011–2012) (39). This direction is expected, because our denominator is restricted to cesarean deliveries and cesarean delivery is an established risk factor for maternal sepsis (16), whereas the comparator studies include all deliveries (roughly one-third of which are cesarean in the United States (2). Differences in case definition (ICD-10-CM vs. ICD-9-CM codes; whether organ dysfunction was required), in the ascertainment window (delivery hospitalization only vs. up to 42 days after discharge) and in coding practice over time may also contribute, and cesarean-specific sepsis rates from other high-income countries based on comparable data are scarce, which precludes a fully denominator-matched comparison.

Our results also extend prior U.S. trends by providing year-by-year estimates for the most recent period. Hirai et al. (18) and Kuklina et al. (8) reported that severe maternal morbidity rose substantially during the 2008–2019 period and that infection-related indicators were a major driver; our data show that the upward trajectory in sepsis specifically has continued through 2022. The modest single-year dip in 2021 (0.122%, vs. 0.130% in 2020) is consistent with the documented disruption of elective obstetric admissions and reductions in overall CS volumes during the COVID-19 pandemic, and the rebound in 2022 suggests this was a transient pandemic-related effect rather than a reversal of the secular trend. We did not have data to distinguish whether the rising prevalence reflects a true increase in disease occurrence (driven by older and more comorbid parturients), improved ascertainment, or both.

### Comparison with global and low- and middle-income country estimates

Estimates from low- and middle-income countries (LMICs) are presented separately because differences in health-system capacity, access to surgical and critical care, case definitions and surveillance make a direct numerical comparison with our U.S. estimates uninformative; the contrast below is therefore descriptive and is not used as a benchmark for our results. Pooled data show a steep gradient across regions: a meta-analysis estimated a global maternal sepsis incidence of 13.16 per 10,000 pregnancies, ranging from 6.31 per 10,000 in the Region of the Americas (a mixed-income region) to 129.17 per 10,000 in the African region (16); a pooled analysis of predominantly facility-based studies in sub-Saharan Africa reported a maternal sepsis prevalence of 19.2%, with very high heterogeneity and risk of bias (40); and a single-center cohort from China, an upper-middle-income country, reported a post-cesarean sepsis incidence of 0.48%–1.12% (1). These figures are roughly one to two orders of magnitude higher than ours, but they derive largely from clinical rather than administrative case definitions, from facility-based rather than nationally representative denominators, and from settings with more limited access to prophylactic antibiotics and intensive care. Outcomes show the same gradient: in the WHO Global Maternal Sepsis Study (GLOSS), intrahospital case fatality among women with infection-related severe maternal outcomes (death or near miss) was 15% (12/81) in low-income, 7% (13/179) in lower-middle-income and 1% (1/91) in upper-middle-income countries, whereas no maternal deaths were reported in high-income countries (41). Our 2.4% in-hospital case fatality should therefore be interpreted against the high-income benchmarks above, not against LMIC series. Although our data cannot address LMIC populations directly, they provide a contemporary high-resource reference against which disparities in maternal sepsis burden and outcomes (32) can be judged, and they show that post-cesarean sepsis remains an important cause of morbidity even where resources are abundant.

### In-hospital mortality, length of stay*,* hospitalization costs and resource utilization

In-hospital mortality among septic patients was 2.4% in our cohort, compared with 0.0% in matched non-septic patients. Among high-income comparators, this case fatality lies between the 1.4% (5/365) reported for severe maternal sepsis in the UK national surveillance study (39) and the approximately 9% (1 in 11) in-hospital case fatality reported in an earlier NIS analysis of delivery-associated sepsis (2002–2015), which also documented a declining trend (37). This spread reflects differences in case definition (severe sepsis requiring organ dysfunction vs. a broader administrative definition that also captures milder presentations coded as sepsis), study period, and the availability of modern resuscitation, intensive care and source-control interventions in high-resource centers. Case fatality in LMIC settings [7%–15% among women with infection-related severe maternal outcomes in lower-middle- and low-income countries in GLOSS (41)] is discussed separately above and is not used as a benchmark here. However, a 2.4% case-fatality rate among obstetric patients hospitalized for delivery remains clinically significant in absolute terms, particularly given the relatively young age and the otherwise low baseline mortality of this population. The doubling of median LOS (6 vs. 3 days) is consistent with prior reports of prolonged hospitalization after surgical sepsis (30, 31) and represents both the time required for acute management (intravenous antibiotics, intensive monitoring, source control) and recovery from associated complications. Together, these findings establish that even in a high-resource healthcare system, post-cesarean sepsis imposes a substantial morbidity burden and substantial resource utilization.

The median total hospitalization cost of USD 71,466 among septic patients—approximately 2.6-fold higher than the median of USD 27,063 among matched non-septic patients—translates into an absolute excess of approximately USD 44,000 per affected hospitalization. This difference likely reflects a combination of a doubling of LOS (with attendant room-and-board and nursing costs), greater use of intensive care, vasopressors, mechanical ventilation, blood products and renal-replacement therapy (reflected in our observed ∼18% transfusion rate among septic patients), higher rates of operative re-intervention for source control, and longer post-acute and rehabilitation needs. The order of magnitude is consistent with U.S. sepsis cost-of-illness estimates of USD 20 billion per year and with surgical-sepsis-specific cost analyses that report per-case excess charges of USD 30,000–60,000 (30, 31). These figures also identify post-cesarean sepsis as a candidate for value-based payment and quality-improvement interventions in obstetric care.

### Patient-, hospital-level factors, and perioperative complications associated with sepsis

In our multivariable analysis, non-elective admission was strongly associated with sepsis—an intuitive finding, since unscheduled CS is typically performed for urgent obstetric complications (e.g., fetal distress, placenta previa, suspected abruption) that themselves increase infection risk through prolonged rupture of membranes, multiple vaginal examinations, chorioamnionitis and emergency surgical technique (9, 42, 43). Higher comorbidity burden, particularly ≥ 3 chronic conditions, was associated with an approximately nine-fold adjusted odds of sepsis, consistent with prior evidence that conditions including severe anemia, hypoproteinemia, malnutrition, diabetes and obesity increase infection risk after CS (1, 44–47). Treatment in teaching hospitals was associated with modestly higher sepsis, plausibly reflecting case-mix (tertiary referral of higher-risk pregnancies) rather than quality-of-care differences, but we cannot exclude ascertainment bias because NIS records more diagnoses in teaching settings. Asian/Pacific Islander race was associated with higher sepsis, which warrants dedicated investigation of genetic, dietary and access-to-care determinants but may also reflect residual confounding by unmeasured clinical or socioeconomic factors.

Our data suggest that post-cesarean sepsis is best understood as a multisystem syndrome. The very large adjusted odds of pulmonary embolism, deep-vein thrombosis and postoperative shock are biologically plausible and consistent with the recognized pathophysiology of sepsis-induced coagulopathy (19–22, 48), endothelial injury (23–25) and microcirculatory failure (49, 50). Pregnancy itself confers a 4–5-fold increase in VTE risk; in the setting of sepsis-associated endothelial dysfunction, hypercoagulability and relative immobilization, the VTE risk is plausibly amplified by an order of magnitude, consistent with our adjusted estimates. The strong association with pulmonary embolism in particular has immediate clinical implications: in any postpartum patient with sepsis, VTE prophylaxis adequacy, point-of-care ultrasound screening for proximal DVT, and a low threshold for cross-sectional imaging of the chest should be considered. The associations with urinary tract infection, chronic respiratory disease and congestive heart failure further suggest that pre-existing or co-incident organ dysfunction amplifies sepsis risk and severity, in line with the established role of endothelial and microvascular dysfunction in sepsis-induced organ failure (23–25, 52, 53). The association with postpartum hemorrhage likely reflects coagulation dysfunction and uterine atony in the setting of systemic infection, but we cannot exclude residual confounding by indication, since patients undergoing emergency CS for obstetric complications are at higher baseline risk of both sepsis and hemorrhage. Although the magnitude of the odds ratios is large, the relatively wide confidence intervals (particularly for rare complications such as PE) emphasize that these are imprecise estimates of association from a large cohort but should be interpreted alongside clinical context.

Finally, septic patients had a markedly higher prevalence of fluid and electrolyte disorders, chronic respiratory disease, and blood transfusion. These findings reflect the systemic nature of sepsis and are consistent with the proposed cascade of sepsis pathophysiology: capillary leak and renal tubular injury drive electrolyte derangement (28, 29); systemic inflammation and ICU-acquired weakness prolong recovery and contribute to chronic respiratory complications (26, 27); and coagulopathy, surgical bleeding and marrow suppression increase transfusion requirements (12, 13). In aggregate, these complications are not isolated events but pathophysiologically intertwined manifestations of sepsis—and together sustain the longer LOS and higher costs observed in the matched comparison.

### Strengths and limitations

A key strength of this study is its use of a large, nationally representative database, which enhances statistical precision and minimizes single-center selection bias.

Several limitations should be noted. First, we required both a cesarean procedure code and a sepsis diagnosis code in the same hospitalization to maximize specificity; however, some A41.x entries may capture non-infectious systemic inflammatory responses. Second, the lack of long-term follow-up restricts outcomes to in-hospital mortality, underscoring the need for health-system surveillance improvements and community-based studies. Third, laboratory data (e.g., inflammatory markers, oxidative stress indicators) were unavailable for mechanistic exploration. Fourth, the NIS lacks critical obstetric variables—emergency vs. elective status, antibiotic prophylaxis details, BMI, labor duration, and chorioamnionitis diagnosis—all established predictors of post-CS infection, which may introduce unmeasured confounding and limit individual risk assessment. Fifth, the cross-sectional, single-hospitalization design precludes temporal sequencing, so all associations are correlational, not causal. Future prospective, multicenter studies incorporating these variables are needed to refine predictive models and risk stratification. Sixth, given the observational and cross-sectional nature of the data, no causal inferences can be made. All reported associations should be interpreted as correlational, and the associated complications may represent either causes or consequences of sepsis, or both (35). Seventh, published estimates from other health systems differ from ours in denominators (all deliveries vs. cesarean deliveries only), case definitions and ascertainment windows, and cesarean-specific benchmarks from comparable high-income systems are scarce; our comparisons with high-income countries are therefore indicative rather than formally standardized, and LMIC data are described separately for context only.

### Implications for clinical practice and health policy

Risk-triggered prevention pathway: non-elective admission and a high comorbidity burden (≥3 conditions) should trigger an enhanced-prevention pathway at admission, comprising weight-based cefazolin (2 g; 3 g if ≥120 kg) within 60 min before skin incision with adjunctive azithromycin for patients in labor or with ruptured membranes (54), chlorhexidine–alcohol skin antisepsis (55), perioperative glycemic control and normothermia, and a documented VTE risk assessment with risk-based pharmacologic prophylaxis, given the very large odds of pulmonary embolism and deep-vein thrombosis (12, 56). Recognition and rescue: obstetric units should adopt the Maternal Early Warning Criteria (57) with mandatory bedside escalation, together with a sepsis response pathway (lactate, blood cultures and broad-spectrum antibiotics within 1 h of recognition of suspected sepsis or shock) aligned with Society for Maternal-Fetal Medicine and Surviving Sepsis Campaign guidance (58). Post-sepsis care: every patient with sepsis should receive a structured multidisciplinary review (obstetrics, anesthesiology, critical care, pharmacy) and reassessment for VTE, respiratory and fluid/electrolyte complications, which were among the complications most strongly associated with sepsis in our data.

Our findings have concrete implications for obstetric care and health policy. At the patient level, the patient-level factors we identified (non-elective admission, higher comorbidity burden, Asian/Pacific Islander race, teaching hospital, “other” payer) can be used to flag high-risk patients before CS for targeted surveillance, intra-operative infection-prevention bundles (timely prophylactic antibiotics, surgical-site preparation, glycemic control, normothermia, VTE prophylaxis) (12, 56) and post-operative enhanced monitoring. At the hospital level, post-cesarean sepsis should be incorporated into obstetric early-warning systems, and institutions should audit infection rates and provide feedback to surgical teams—measures that have been shown to reduce sepsis-related mortality in non-obstetric populations (31). At the health-system level, given the approximately 2.6-fold higher median costs observed here, post-cesarean sepsis is a strong candidate for value-based care interventions and for inclusion in standard hospital-acquired condition reduction programs. Finally, prospective research priorities include: development and validation of risk-prediction models calibrated to contemporary U.S. obstetric populations; evaluation of prevention bundles using registries with biomarker and process-of-care data; cost-effectiveness analyses of prevention bundles; and investigation of the apparent racial disparity in sepsis odds, which may reflect access-to-care or socioeconomic determinants that warrant targeted intervention.

## Conclusions

In this national analysis of 1.59 million U.S. cesarean deliveries (2016–2022), post-cesarean sepsis prevalence rose 2.3-fold. Though rare, sepsis was independently linked to markedly higher in-hospital mortality (2.4% vs. 0.0%). doubled hospital stay, 2.6-fold higher costs, and associated with PE, DVT, shock, UTI, and hemorrhage. These findings mark post-cesarean sepsis as a rising public health concern and support: integrating sepsis into obstetric early-warning and post-op surveillance, implementing prevention bundles (antibiotics, site prep, glycemic control, VTE prophylaxis), and prospective research on risk-stratified, value-based interventions.

## Data Availability

Publicly available datasets were analyzed in this study. The data can be obtained from the Healthcare Cost and Utilization Project (HCUP) Nationwide Inpatient Sample (https://hcup-us.ahrq.gov/nisoverview.jsp) under a signed Data Use Agreement; the authors are not permitted to redistribute them.
