# Supplementary material for: Trends, outcomes and patient- and hospital-level factors associated with sepsis following cesarean delivery: a propensity-matched nationwide inpatient analysis (2016–2022)
Source: Front Surg. 2026 Oct 21;13:1871936. doi: 10.3389/fsurg.2026.1871936 (PMC13731296; doi:10.3389/fsurg.2026.1871936)
Supplement: Supplementary file 1 [file Table1.docx]

**Table S1. ICD-10 codes used to identify sepsis and cesarean section.** Sepsis was defined using ICD-10-CM diagnosis codes, and cesarean section using ICD-10-PCS procedure codes.

| **Variable** | **ICD-10-CM Codes** |
| --- | --- |
| Sepsis | A40.x, A41.x, R65.20, R65.21 |
| Cesarean section | 10D00Z0, 10D00Z1, 10D00Z2 |

*Abbreviations:* ICD-10-CM, International Classification of Diseases, 10th Revision, Clinical Modification; ICD-10-PCS, International Classification of Diseases, 10th Revision, Procedure Coding System.
