# Supplementary material for: Trends, outcomes and patient- and hospital-level factors associated with sepsis following cesarean delivery: a propensity-matched nationwide inpatient analysis (2016–2022)
Source: Front Surg. 2026 Oct 21;13:1871936. doi: 10.3389/fsurg.2026.1871936 (PMC13731296; doi:10.3389/fsurg.2026.1871936)
Supplement: Supplementary file 2 [file Table2.docx]

**Table S2. ICD-10-CM diagnosis codes for the types of bacterial sepsis included in this study.** Types include other sepsis, streptococcal sepsis, and severe sepsis with or without septic shock.

| **Variable** | **ICD-10-CM Codes** |
| --- | --- |
| **other sepsis** | A41.x |
| **streptococcal sepsis** | A40.x |
| **severe sepsis without septic shock** | R65.20 |
| **severe sepsis with septic shock** | R65.21 |

*Abbreviations:* ICD-10-CM, International Classification of Diseases, 10th Revision, Clinical Modification.
