# Supplementary material for: Trends, outcomes and patient- and hospital-level factors associated with sepsis following cesarean delivery: a propensity-matched nationwide inpatient analysis (2016–2022)
Source: Front Surg. 2026 Oct 21;13:1871936. doi: 10.3389/fsurg.2026.1871936 (PMC13731296; doi:10.3389/fsurg.2026.1871936)
Supplement: Supplementary file 3 [file Table3.docx]

**Table S3. Variables included in the binary logistic regression analysis.** Variables are grouped into patient demographics, hospital characteristics, and comorbidities.

| **Variables Categories** | **Specific Variables** |
| --- | --- |
| **Patient demographics** | Age (18-24 years, 25-29 years, 30-34 years and ≥35 years), race (White, Black, Hispanic, Asian or Pacific Islander, Native American and Other)，Number of Comorbidity |
| **Hospital characteristics** | Type of admission (non-elective, elective), bed size of hospital (small, medium, large), teaching status of hospital (nonteaching, teaching), location of hospital (rural, urban), type of insurance (Medicare, Medicaid, private insurance, self-pay, no charge, other), location of the hospital (northeast, Midwest or north central, south, west) |
| **Comorbidities** | AIDS, Alcohol abuse, Deficiency anemias, Rheumatoid arthritis, Congestive heart failure, Dementia, Coagulopathy, Depression  Diabetes, Drug abuse, Hypertension, Liver disease, Lymphoma, Fluid and electrolyte disorders, Metastatic cancer, Obesity, Paralysis, Peripheral vascular disorders, Psychoses, Pulmonary circulation disorders, Renal failure, Smoking, Solid tumor without metastasis, Peptic ulcer disease excluding bleeding and Valvular disease |

*Abbreviations:* AIDS, acquired immunodeficiency syndrome.
