# Supplementary material for: Trends, outcomes and patient- and hospital-level factors associated with sepsis following cesarean delivery: a propensity-matched nationwide inpatient analysis (2016–2022)
Source: Front Surg. 2026 Oct 21;13:1871936. doi: 10.3389/fsurg.2026.1871936 (PMC13731296; doi:10.3389/fsurg.2026.1871936)
Supplement: Supplementary file 4 [file Table4.docx]

**Table S4. Univariate and multivariable logistic regression analyses of preoperative comorbidities associated with sepsis after cesarean section in the matched cohort.** Data are presented as n (%). Results of the multivariable analysis are presented as odds ratios (OR) with 95% confidence intervals (CI).

| **Comorbidities** | | **Univariate Analysis** | | | **Multivariate Logistic Regression** | | |
| --- | --- | --- | --- | --- | --- | --- | --- |
|  |  | **Matched-No Sepsis** | **Sepsis** | **P** | **OR** | **95% CI** | **P** |
| **Preoperative comorbidities** | |  |  |  |  |  |  |
|  | Acquired immune deficiency syndrome | 1 (0.0%) | 5 (0.3%) | <0.001 | 1.573 | 0.062-39.951 | 0.784 |
|  | Alcohol abuse | 4 (0.0%) | 3 (0.2%) | 0.176 | 3.712 | 0.444-31.023 | 0.226 |
|  | Deficiency anemias | 213 (2.5%) | 113 (6.7%) | <0.001 | 0.956 | 0.500-1.827 | 0.891 |
|  | Rheumatoid arthritis | 21 (0.3%) | 14 (0.8%) | <0.001 | 1.810 | 0.656-4.993 | 0.252 |
|  | Congestive heart failure | 8 (0.1%) | 58 (3.5%) | <0.001 | 3.981 | 1.346-11.774 | 0.013 |
|  | Coagulopathy | 158 (1.9%) | 150 (9.0%) | <0.001 | 1.315 | 0.695-2.486 | 0.400 |
|  | Depression | 358 (4.3%) | 93 (5.6%) | 0.021 | 0.472 | 0.246-0.905 | 0.024 |
|  | Diabetes | 176 (2.1%) | 41 (2.4%) | 0.373 | 0.591 | 0.294-1.188 | 0.140 |
|  | Drug abuse | 196 (2.3%) | 112 (6.7%) | <0.001 | 0.906 | 0.477-1.722 | 0.764 |
|  | Hypertension | 408 (5.6%) | 160 (11.6%) | <0.001 | 0.823 | 0.452-1.498 | 0.524 |
|  | Liver disease | 50 (0.6%) | 52 (3.1%) | <0.001 | 1.361 | 0.604-3.066 | 0.457 |
|  | Fluid and electrolyte disorders | 83 (1.0%) | 565 (33.7%) | <0.001 | 19.06 | 10.542-34.464 | <0.001 |
|  | Obesity | 1342 (18.6%) | 279 (20.3%) | 0.140 | 0.458 | 0.258-0.813 | 0.008 |
|  | Paralysis | 2 (0.0%) | 7 (0.4%) | <0.001 | 3.011 | 0.360-25.183 | 0.309 |
|  | Peripheral vascular disorders | 4 (0.0%) | 17 (1.0%) | <0.001 | 5.475 | 1.325-22.623 | 0.019 |
|  | Psychoses | 681 (8.1%) | 227 (13.6%) | <0.001 | 0.701 | 0.386-1.276 | 0.245 |
|  | Pulmonary circulation disorders | 7 (0.1%) | 36 (2.1%) | <0.001 | 0.612 | 0.15-2.492 | 0.493 |
|  | Renal failure | 15 (0.2%) | 18 (1.1%) | <0.001 | 0.705 | 0.203-2.457 | 0.583 |
|  | Valvular disease | 11 (0.1%) | 9 (0.5%) | 0.002 | 0.886 | 0.200-3.925 | 0.874 |

*Abbreviations:* OR, odds ratio; CI, confidence interval.
