# Supplementary material for: Trends, outcomes and patient- and hospital-level factors associated with sepsis following cesarean delivery: a propensity-matched nationwide inpatient analysis (2016–2022)
Source: Front Surg. 2026 Oct 21;13:1871936. doi: 10.3389/fsurg.2026.1871936 (PMC13731296; doi:10.3389/fsurg.2026.1871936)
Supplement: Supplementary file 5 [file Table5.docx]

**Table S5. Univariate and multivariable logistic regression analyses of complications associated with sepsis after cesarean section in the matched cohort.** Data are presented as n (%). Results of the multivariable analysis are presented as odds ratios (OR) with 95% confidence intervals (CI).

| **Complications** | | **Univariate Analysis** | | | **Multivariate Logistic Regression** | | |
| --- | --- | --- | --- | --- | --- | --- | --- |
|  |  | **Matched-No Sepsis** | **Sepsis** | **P** | **OR** | **95% CI** | **P** |
|  | Chronic respiratory diseases | 4 (0.0%) | 19 (1.1%) | <0.001 | 13.162 | 2.926-59.205 | 0.001 |
|  | Urinary tract infection | 18 (0.2%) | 72 (4.3%) | <0.001 | 25.532 | 12.658-51.5 | <0.001 |
|  | Postpartum depression | 13 (0.2%) | 11 (0.7%) | <0.001 | 2.080 | 0.657-6.584 | 0.213 |
|  | Postpartum hemorrhage | 360 (4.3%) | 250 (14.9%) | <0.001 | 2.358 | 1.829-3.040 | <0.001 |
|  | Amniotic fluid embolism | 1 (0.0%) | 7 (0.4%) | <0.001 | 0.818 | 0.040-16.872 | 0.897 |
|  | Pulmonary embolism | 1 (0.0%) | 30 (1.8%) | <0.001 | 61.681 | 6.714-566.627 | <0.001 |
|  | Blood transfusion | 201 (2.4%) | 298 (17.8%) | <0.001 | 3.846 | 2.897-5.106 | <0.001 |
|  | Postoperative shock | 3 (0.0%) | 66 (3.9%) | <0.001 | 28.650 | 6.251-131.306 | <0.001 |
|  | DVT | 3 (0.0%) | 27 (1.6%) | <0.001 | 24.638 | 4.680-129.716 | <0.001 |
|  | Nausea | 31 (0.4%) | 10 (0.6%) | 0.184 | 0.792 | 0.245-2.561 | 0.697 |

*Abbreviations:* OR, odds ratio; CI, confidence interval; DVT, deep vein thrombosis.
