# Supplementary material for: Trends, outcomes and patient- and hospital-level factors associated with sepsis following cesarean delivery: a propensity-matched nationwide inpatient analysis (2016–2022)
Source: Front Surg. 2026 Oct 21;13:1871936. doi: 10.3389/fsurg.2026.1871936 (PMC13731296; doi:10.3389/fsurg.2026.1871936)
Supplement: Supplementary file 6 [file Table6.docx]

**Table S6. Annual incidence of sepsis after cesarean section, 2016–2022.** Incidence is expressed as a percentage of all cesarean deliveries in each year.

| **Year** | | **Annual incidence (%)** |
| --- | --- | --- |
|  | 2016 | 0.058 |
|  | 2017 | 0.075 |
|  | 2018 | 0.103 |
|  | 2019 | 0.119 |
|  | 2020 | 0.130 |
|  | 2021 | 0.122 |
|  | 2022 | 0.136 |
